# Mitochondrial Dysfunction Contributes to the Pathogenesis of Alzheimer's Disease

**DOI:** 10.1155/2015/509654

**Published:** 2015-06-29

**Authors:** Fabian A. Cabezas-Opazo, Katiana Vergara-Pulgar, María José Pérez, Claudia Jara, Cesar Osorio-Fuentealba, Rodrigo A. Quintanilla

**Affiliations:** ^1^Laboratory of Neurodegenerative Diseases, Centro de Investigación Biomédica, Universidad Autónoma de Chile, San Miguel, 8900000 Santiago, Chile; ^2^Departamento de Kinesiología, Universidad Metropolitana de Ciencias de la Educación, Ñuñoa, 7760197 Santiago, Chile

## Abstract

Alzheimer's disease (AD) is a neurodegenerative disease that affects millions of people worldwide. Currently, there is no effective treatment for AD, which indicates the necessity to understand the pathogenic mechanism of this disorder. Extracellular aggregates of amyloid precursor protein (APP), called A*β* peptide and neurofibrillary tangles (NFTs), formed by tau protein in the hyperphosphorylated form are considered the hallmarks of AD. Accumulative evidence suggests that tau pathology and A*β* affect neuronal cells compromising energy supply, antioxidant response, and synaptic activity. In this context, it has been showed that mitochondrial function could be affected by the presence of tau pathology and A*β* in AD. Mitochondria are essential for brain cells function and the improvement of mitochondrial activity contributes to preventing neurodegeneration. Several reports have suggested that mitochondria could be affected in terms of morphology, bioenergetics, and transport in AD. These defects affect mitochondrial health, which later will contribute to the pathogenesis of AD. In this review, we will discuss evidence that supports the importance of mitochondrial injury in the pathogenesis of AD and how studying these mechanisms could lead us to suggest new targets for diagnostic and therapeutic intervention against neurodegeneration.

## 1. Introduction

Clinically, AD is characterized by a progressive memory and cognitive impairment that gradually compromise entire brain health of the patients [1, 2]. AD is characterized by the presence of two major groups of protein aggregates: (1) senile plaques and (2) NFTs [1, 2]. Senile plaques majorly contain glial cells and aggregates of A*β* peptide (A*β*_1–40_ and A*β*_1–42_). (2) NFTs are intraneuronal structures formed by tau protein in a hyperphosphorylated form [1, 2]. A*β*_1–40_ and A*β*_1–42_ derived from the partial processing of amyloid precursor protein (APP) in neurons and glial cells, which induced neuronal injury, inflammation, and oxidative stress [1, 2]. At the same time, tau pathology has a profound effect on neuronal health, affecting different processes such as transport, autophagy, and neuronal communication [2], and recent studies suggest a major role in the progression of AD [3]. In this context, important evidence has suggested that A*β* and tau pathology can affect mitochondrial function in brain cells [4]. Mitochondria are responsible for energy supply, detoxification, and communication in brain cells and accumulative evidence suggests that they could have a role in the pathogenesis of AD [5]. In AD, mitochondrial function could be compromised in three different aspects: (1) morphology or mitochondrial dynamics, (2) bioenergetics, and (3) transport.

1. Defects in mitochondrial dynamics are related to changes in mitochondrial fission/fusion proteins such as dynamin-related protein-1 (Drp1), Mitofusins 1 and 2 (Mfn1 and Mfn2), and optic atrophy protein (OPA-1) [6]. Mfn1 and Mfn2 are GTPases that regulate mitochondrial fusion, followed by fusion of the inner membranes mediated by OPA1 for a mechanism of ubiquitination and subsequent proteasomal degradation [6, 7]. Drp1 is also a GTPase that participates in mitochondrial fission (elongation) and is predominantly locating in the cytoplasm [6–8]. Overall, fine regulation of fission and fusion proteins is necessary to maintain a normal mitochondrial function (energy supply, antioxidant defenses, and calcium homeostasis) in brain cells [6–8]. In this review, we discuss evidence of mitochondrial fission/fusion defects in neurodegenerative diseases, principally in AD.
2. Defects in mitochondrial bioenergetics in AD are extending to a decrease in ATP production, impairment of electron transfer system (ETS), mitochondrial depolarization, and increase of reactive oxygen species (ROS) production [9]. ETS is responsible for oxidative phosphorylation, which is the biochemical pathway that produces ATP by consuming oxygen [9, 10]. In ETS, the electrons are sequentially transferred from respiratory complexes I to complex IV [10]. As a consequence, an electrochemical proton gradient is building across the inner mitochondrial membrane, and this force produces ATP by complex V [10]. This highly regulated process is affecting by oxidative stress and calcium overload leading to neurodegeneration in the AD brain [9].
3. Defects in mitochondrial transport regularly affect neuronal function including autophagy and neuronal communication and finally could induce synaptic loss [11–13]. These effects have been observed in different cellular and mice models used to replicate AD pathology [11–13] and represent an important factor in the progression of AD. In this review, we will discuss evidence in which mitochondrial transport impairment is contributing to neurodegeneration in AD.

**Figure 1:**
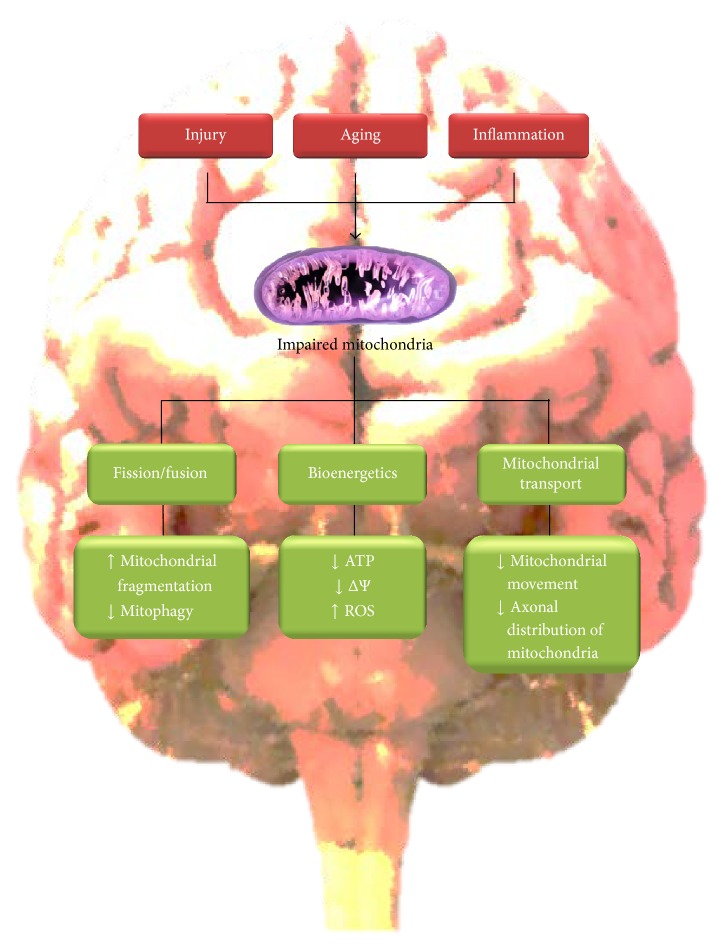
Mitochondrial dysfunction in AD. Factors that contributed to AD such as injury, aging, and inflammation can affect three critical aspects of mitochondrial function: (1) mitochondrial dynamics (inducing fragmentation), (2) bioenergetics (ATP and ROS production), and (3) mitochondrial movement (synaptic function). ATP: adenosine triphosphate; ΔΨ: mitochondrial membrane potential; ROS: reactive oxygen species.

Overall, in this review, we will discuss relevant studies that suggest a role of mitochondrial injury in AD (Figure 1). Mitochondrial impairment could contribute to neurodegeneration in AD and the improvement of mitochondrial health could be considered a serious new target of therapeutic intervention against AD.

## 2. Mitochondrial Fission/Fusion Cycle in AD

### 2.1. General Considerations

Mitochondria are a dynamic organelle, which interacts with each other forming an intricate network similar to the endoplasmic reticulum (ER) [7]. Mitochondria pass through several processes of fission and fusion (shortened and elongation) called fission/fusion cycle or “mitochondrial dynamics” [7, 8]. This process consists of contact-dependent fusion events, in which moving mitochondria make contact with a passive one and turn back fusing side to side, forming the tubular mitochondrial network [7, 8]. This process is consecutively followed by a fission event, in which mitochondria are segmented, giving birth to two daughter's mitochondria [8]. One of these daughter's mitochondria undergoes another fusion event while the other depolarized mitochondria go through autophagy [8, 14–16]. The fission/fusion cycle determines mitochondrial morphology [8, 14, 16], as well as the control of mitochondrial integrity and functionality [7, 14, 17]. Interestingly, a role of this process in embryonic development [18] and cell survival during stress conditions has been described [19].

Five main proteins are controlling mitochondrial dynamics: Drp1, mitochondrial fission protein 1 (Fis1), Opa1, Mfn1, and Mfn2 [20]. Drp1 is locating mainly in the cytoplasm while a little portion is in the mitochondrial outer membrane [20]. Drp1 function is to spin off both mitochondrial membranes, by self-assembling and constricting membranes through its GTPase activity [21, 22]. Fis1 is predominant in mitochondrial outer membrane and has a role in recruiting Drp1 from cytoplasm to mitochondrial outer membrane [23–25]. Opa1 and both Mfn1 and Mfn2 mediate inner and outer membrane fusion, respectively [26, 27]. Opa1 also has a role in mitochondrial quality control, since reduced levels of this protein and its proteolytic processing lead to impaired fusion of mitochondria [14, 28].

### 2.2. Physiological Functions of Mitochondrial Dynamics in Neurons

Mitochondrial dynamics appears to have a role in embryonic survival and neuronal development, since reduction in the expression of Mfn2 gene leads to a decrease in dendrites and spine development of Purkinje cells [29]. In this same model, impaired mitochondrial fission causes neurodegeneration, since mitochondria of Mfn2 null cells exhibit an increased diameter with no mitochondria presence in dendrites [29]. All these abnormalities lead to placental defects with a consequent embryo mortality and cerebellar degeneration in postnatal Mfn2 null mice [29].

Studies of knocking down and overexpression of Drp1, Fis1, Opa1, Mfn1, and Mfn2 resulted in reduced spine density and a lack of mitochondrial content in spines of hippocampal neurons [30]. Interestingly, mitochondrial dynamics defects have been described in astrocytes and glial cells [31]. Astrocytes, exposed to proinflammatory cytokines, increased mitochondria fission followed by a rise of the mitophagy process of dysfunctional mitochondria [31]. These are important observations because neuroinflammation plays a role in the pathogenesis of AD and astrocytes and glial cells actively participate in neuronal communication [31].

### 2.3. Defects of Mitochondrial Dynamics in AD

Analysis of brain samples from AD patients showed altered mitochondrial morphology compared to mitochondria of age-matched individuals [32]. These results were confirmed in a study using M17 neuroblastoma cell line, in which wild-type and Swedish mutant forms of APP protein were overexpressed, leading to changes in mitochondrial structure [33]. Mitochondria morphology changed from thin and elongated to a fragmented and punctiform form, having an effect more severe in cells with the Swedish mutation of APP [33]. In addition, rat hippocampal neurons treated with oligomeric amyloid-*β*-derived diffusible ligands (ADDLs) showed a decrease in the mitochondrial length and a reduction of mitochondrial density in neurites [30]. Complementary studies showed that treatment of hippocampal neurons with A*β*_1–42_ reduced axonal mitochondrial density [34]. A similar observation was made in transgenic mice overexpressing APP/A*β*, in which axonal mitochondria were shorter than wild-type [34]. In this context, a recent study in the cortex of rhesus monkeys showed that mitochondria located at presynaptic region present a donut-like shape while aging [35]. These changes in mitochondrial morphology correlated with an ROS increase and produced memory impairment in aged monkeys [35]. On the other hand, in M17 cells the expression of A*β* reduced the levels of Drp1 and Opa1, while Fis1 levels increased [33]. Further studies that analyzed cortical samples of AD patients showed increased mRNA levels of Drp1 and Fis1, while fusion proteins Opa1, Mfn1, and Mfn2 showed a reduction in mRNA levels [36]. Interestingly, cytosolic fraction obtained from brain samples of AD patients showed a decrease in Drp1 levels [30]. Apparently these observations are explained by an increase of Drp1 associated with mitochondria in AD [30]. Finally, in M17 cells the overexpression of Drp1 induces mitochondrial fragmentation while Opa1 expression induces mitochondrial elongation [30].

In addition, mitochondrial dynamics is affected by calcium overload in neurons [37]. For instance, a treatment of cortical neurons with NMDA and glucose deprivation leads to a reduction of Mfn2 levels [38]. This reduction in Mfn2 levels was sustained over time and correlated with mitochondrial fragmentation and activation of Drp1 that migrated from cytosol to mitochondria [38]. Interestingly, Mfn2 appears to maintain mitochondrial fragmentation even after removal of NMDA, which suggests that Mfn2 has a role in perpetuating neurotoxicity through affecting mitochondrial dynamics [38]. These observations were extended by* in vitro* studies of N2a cells that expressed APP Swedish mutation (N2a-APPswe) [39]. Here, A*β* accumulation induced a decrease in both Mfn1 and Mfn2 levels, with a subsequent fragmentation of mitochondria [39].

Interestingly, important studies have suggested that A*β*-induced neurotoxicity is produced by the direct interaction of A*β* aggregates with the mitochondria [40, 41]. A*β* can be accumulated in the mitochondria by mechanism dependent on the translocase of outer membrane transporter (TOM) [41]. This accumulation affects the functionality of the mitochondria by blocking the entry of cytoplasmic proteins into the mitochondrial matrix [41]. In addition, studies made in N2a cells expressing human mutant APP protein showed that A*β* accumulated in cytosol and mitochondrial membranes of N2a cells that expressed human mutant APP [40]. These results were confirmed in cortical slices from Tg2576 mice, where immunoreactivity against A*β* was also colocalized with mitochondria [40]. Additionally, in cortical neurons of APP/A*β* mice, A*β* was found associated with synaptic mitochondria, suggesting an important role in synaptic neurodegeneration [34]. In addition, in cortical lysates of A*β*/PP1 mice model, immunoprecipitation and immunofluorescence studies showed that A*β* interacts with Drp1 [36]. The same results were obtained in both cortical lysates and cortical sections from brains samples of AD patients [36].

Tau pathology is considered a major player in the pathogenesis of AD [1, 2]. Accumulation of caspase-cleaved and phosphorylated tau in neurons results in the formation of NFTs [1, 42–44]. Interestingly, recent studies have proposed the possible role of tau in mitochondrial dynamics impairment in AD [45]. A recent study in rat cortical cells showed that overexpression of tau decreases mitochondrial motility, with a subsequent shortening of mitochondrial length [45]. Also, transient expression of caspase-cleaved tau in immortalized cortical neurons results in mitochondria being rounded and severely fragmented [46]. Further studies in rat cortical neurons showed the similar results when caspase-cleaved tau was present in these cells [47]. Regarding hyperphosphorylated tau, immunoprecipitation assays of cortical lysates of triple transgenic mice (3xTg-AD) and APP/PS1 mice showed a significant colocalization between hyperphosphorylated tau and Drp1, and these observations were confirmed by double-labeling immunofluorescence in cortical and hippocampal sections [48]. In addition, studies of immunoprecipitation and immunofluorescence demonstrated that hyperphosphorylated tau interacts with Drp1 both in cortical lysates and in sections from AD brains [48]. Further studies confirmed the importance of these observations when mitochondrial morphology was examined in cortical neurons expressing pseudophosphorylated tau at S396/404, which represents tau hyperphosphorylation at PHF-1 residues, which are forming the NFTs [49]. Expression of pseudophosphorylated tau (T42EC) did not affect mitochondrial morphology compared to neurons that expressed GFP and full-length tau [49]. However, T42EC expression enhanced A*β*-induced mitochondrial depolarization and increased superoxide levels compared to mature neurons expressing full-length tau [49]. These results indicate that pathological forms of tau affect mitochondrial dynamics, and these forms can also interact with Drp1 in both transgenic mouse models and AD brain [48]. Further studies are needed to explore if pathological forms of tau affect expression or activity of proteins involved in mitochondrial fission/fusion cycle.

## 3. Bioenergetics Defects of Mitochondria in AD

Accumulative evidence suggests that mitochondrial injury could contribute to the pathogenesis of AD [50–53]. Evidence supporting this hypothesis includes the fact that mitochondria could present defects in the morphology, decrease metabolic activity, and transport impairment [50]. Mitochondria provide ATP and antioxidant with power to prevent neuronal injury in AD [49]. In this context, relevant evidence showed a reduced ATP production, excessive ROS levels, and significant respiratory defects in mitochondrial preparations from several AD mice models [4, 5].

Impaired mitochondrial function is consistent with altered glucose metabolism in AD brain [54]. In addition, studies in mitochondrial preparations from postmortem brain samples of AD patients showed a reduced activity of mitochondrial tricarboxylic acid cycle enzymes [50]. Interestingly, reduced levels of mitochondrial DNA are presented within neurons prior to the formation of NFTs, indicating that mitochondrial injury could be an early neuropathological sign that will contribute to AD [55].

### 3.1. Mitochondrial Dysfunction and A*β* Pathology in AD

Most of the data that showed mitochondrial injury in AD comes from studies in which A*β* directly affected mitochondrial bioenergetics [4, 5]. A*β* has a significant role in the ROS production mechanism in AD (for review see [55]) and several studies showed that direct exposure to A*β* significantly impairs functionality of the mitochondrial ETS [9]. The ETS is central to ATP production, and its constituent enzyme complexes are the source of ROS generation [10]. By exposing isolated mitochondria preparations to different forms of A*β* peptide (mostly A*β*_1–40_ and A*β*_1–42_), several groups showed a significant impairment in the ETS activity [56–59]. These observations indicate that A*β* can effectively affect mitochondrial function through increasing ROS levels in AD [55–59].

Exposure to increased levels of A*β* decreases mitochondrial membrane potential and respiration rates [55–58]. In addition, several groups have reported that A*β* treatment induced mitochondrial swelling, apoptosis, opening of mitochondrial transition pore (mPTP), and increase of ROS production [55, 60–62]. In general, all these effects mediated by A*β* can have a substantial impact not only on mitochondrial functionality, but also on overall cell viability.

In addition, relevant studies explored whether mitochondria-derived ROS have an effect on A*β* generation [63]. Treatment with the respiratory inhibitors, rotenone and antimycin, resulted in mitochondrial injury and enhanced ROS levels [63]. Interestingly, both treatments increased the levels of A*β* and treatment with an antioxidant prevented mitochondrial dysfunction and reduced formation of A*β* in neuronal cells [63]. In addition, cells that overexpressed A*β* showed an impaired mitochondrial respiration, altered mitochondrial morphology, and reduced mitochondrial transport [63]. These observations suggest that mitochondria-derived ROS are capable of increasing A*β* production* in vitro* and* in vivo*, an effect that could contribute to the pathogenesis of AD.

Others studies examined A*β*-mediated mitochondrial failure in different AD mice models [9, 64]. For example, Xie et al. [64] using multiphoton microscopy studied mitochondrial structural and functional changes in AD mouse models [64]. These studies showed depolarized and fragmented mitochondria in the vicinity of A*β* plaques in APP/PS1 transgenic mouse brain [64]. In addition, the neuronal population that showed oxidative stress presented mitochondrial depolarization in mice that express both mutant human APP and PS1 (APP/PS1) [55, 64]. Complementary studies showed significant changes in mitochondrial morphology, loss of the integrity of synaptic mitochondria, and reduced ATP production in brain samples of APP/PS1 mice [65]. These observations indicate that the presence of A*β* aggregates can act as source of toxicity inducing morphology and functional abnormalities in mitochondria [65].

### 3.2. Mitochondrial Impairment and Tau Pathology in AD

Mitochondrial dysfunction may be fundamental to the pathogenesis of AD [49, 52, 66]. These observations include altered mitochondrial morphology, depressed metabolic activity, and release of proapoptotic proteins in both animal models and neuronal cells [53, 67, 68]. AD mice that express pathological forms of tau showed mitochondrial impairment in different brain areas [66]. For instance, brain samples from transgenic pR5 mice, a mouse that overexpressed the mutant P301 of tau protein, showed a decrease of mitochondrial complexes activity [69]. P301S mice overexpress the human tau mutated gene, resulting in tau hyperphosphorylation and NFTs formation [1, 2]. Mitochondrial samples from pR5 mice showed mitochondrial depolarization, impaired respiration, and high ROS levels [69]. In addition, mitochondrial dysfunction was observed in 3xTg-AD at three months of age that means prior to the development of amyloid plaque [68]. Brain samples from 3xTg-AD showed mitochondrial impairment, with a decrease in mitochondrial respiration, and pyruvate dehydrogenase (PDH) activity as early as three months of age [68]. 3xTg-AD mice also exhibited increased oxidative stress as was observed by an increase in hydrogen peroxide production and lipid peroxidation [68]. These observations are important because this transgenic mouse contains mutations in three genes (human APPswe, TauP301L, and PS1M146V genes), which present neurodegenerative changes similar to AD and the frontotemporal dementia (FTD) [1, 2]. Interestingly, mitochondrial dysfunction was also found in another AD triple transgenic mouse [53]. These mice came from the crossing of P301L tau transgenic mice with APPswPS2N141l double transgenic mice, which presented evident mitochondrial dysfunction before the development of amyloid pathology [53]. Proteomic studies using this mouse showed an altered expression of mitochondrial complexes I and IV [53]. Additionally, these mice showed mitochondrial depolarization, reduced ATP synthesis, and increased ROS production [53]. Based on this evidence, the genomic regulation of mitochondrial proteins induced by pathological forms of tau or A*β* may play a crucial role in the pathogenesis of AD.

## 4. Mitochondrial Movement Defects in AD

### 4.1. Axonal Transport of Mitochondria

Mitochondrial movement in the axon is possible by the action of microtubules [11]. They transport mitochondria between the soma and the nerve terminals with the aid of different proteins complexes [11–13]. There are two types of axonal transport: (i) slow, for moving cytoplasmic and cytoskeletal proteins and (ii) fast, for moving membrane-bounded organelles (MBOs) including vesicles and mitochondria [13]. Moreover, transport of molecules to the nerve terminals is called “anterograde transport,” and the movement through the soma is called “retrograde transport” [11–13].

Anterograde transport is performed by kinesin-1 protein (KIF5) [11, 12], which is a heterotetramer formed by two-kinesin heavy chain (KHC) and two-kinesin light chain (KLC) [11–13, 70–72]. In mammals are three isoforms of KIF5 (KIF5A, KIF5B, and KIF5C), of which KIF5A and KIF5C are expressed selectively in neurons [11–13, 70–72]. KIF5 has an aminoterminal motor domain with ATPase (that moves toward the plus end of the microtubule) and a C-terminal tail (for binding cargo adapter) [11–13, 70–72]. The Milton protein (in* Drosophila*) or its orthologous in mammalian (Trak1 and Trak2) acts as motor adaptor for KIF5 with mitochondrial receptor Miro (in* Drosophila*) or Miro1 and Miro2 (in mammals) [70, 71]. On the other hand, dynein proteins are responsible for the retrograde transport [11, 70–72]. This protein complex has two heavy chains (DHC), an intermediate (DIC), a light intermediate (DLIC), and a group of light chains (DLC) [11, 70, 71]. The DHC has a globular motor domain that can exhibit ATPase activity and bind to microtubules [73]. Moreover, dynein activity depends on its interaction with the dynactin complex [11, 12, 70, 72–74]. Dynactin contains a rod domain for cargo binding and a projecting arm with microtubule-binding sites linking cytoplasmic dynein to its cargo (DLIC and DLC) [73, 75]. Finally, neurons require stationary mitochondria for dissociating mitochondria from motor proteins or anchoring mitochondria to the cytoskeleton [74–76]. Recently syntaphilin (SNPH) was identified, a protein that produces the docking/retaining of mitochondria in axons [74, 75]. SNPH acts as a “static anchor” for axonal mitochondria [74–76]. SNPH targets axonal mitochondria through its C-terminal mitochondria-targeting domain and axon-sorting sequence [70, 75, 76].

### 4.2. Defects in Axonal Transport and Mitochondrial Transport in AD

Tau localizes predominantly in axons, where it regulates microtubule dynamics, neuronal polarity, and axonal stability [77, 78] and contributes to the axonal transport of organelles to nerve terminals [79]. Mitochondrial population is reduced in cultured neurons from different animal models of AD [78]. Studies in 3xTg-AD mice showed deficits in axonal transport and axonal swelling that precede A*β* deposition or filamentous tau aggregation, suggesting that such deficits might be early events in AD [73].

Studies in mouse hippocampal neurons treated with A*β* peptide showed a significant reduction in the anterograde mitochondrial transport [80]. In addition, A*β* treatment reduced mitochondria length and decreased the expression of synaptophysin (a presynaptic protein), indicating that A*β* could affect synaptic process through mitochondrial injury [80]. In general, reductions in mitochondria and/or the anterograde transport of mitochondria are likely responsible for the synaptic failure, which may cause memory impairment in AD [80]. Another study in hippocampal neurons from wild-type and tau-deficient mice demonstrated that the exposure of neurons to A*β* inhibited axonal mobility of mitochondria and/or neurotrophin receptor TrkA in wild-type neurons [81]. The effects observed were stronger on anterograde transport, and the complete or partial tau reduction prevented these defects [81]. Also tau levels were more critical for axonal transport in the presence of A*β*, suggesting that A*β* requires tau to impair axonal transport, and its reduction protects against A*β*-induced axonal transport defects [81]. In addition, A*β* oligomers impaired axonal transport of cargoes through activation of NMDA receptor, glycogen synthase kinase 3*β* (GSK3*β*), and casein kinase 2 (CK2) [81]. Moreover, primary neurons from transgenic mice expressing AD-linked forms of hAPP showed defects in mitochondrial axonal transport [82]. In addition, reduction of tau expression by genetic ablation or postnatal knockdown prevented mitochondrial transport defects in neurons from hAPP transgenic mice [82]. Finally, it has been reported that depletion of cyclophilin D (CypD), a mitochondrial protein that forms mPTP and induces apoptosis, significantly prevented the defects in mitochondrial transport and dynamics, induced by A*β* treatment of cortical neurons [83].

Furthermore studies* in vitro* by Calkins et al. [84] showed that progressive accumulation of A*β* oligomers affected mitochondrial morphology, reduced anterograde mitochondrial transport, and impaired synaptic activity [84]. At the same time, earlier findings showed impairment of mitochondrial transport and mitochondrial uncoupling in cultured neurons treated with A*β* [84]. This indicates that depolarized mitochondria (impaired) moved in the retrograde direction, and functional mitochondria moved in the opposite direction (anterograde) [84].

Additionally, Llorens-Martín et al. [85] showed that overexpression of GSK3*β* increases the number of mobile mitochondria in the axons, and reduction in GSK3*β* activity produced an increase in mitochondria pausing [85]. Complementary studies showed that reduction of GSK3*β* activity, using a dominant negative (DN-GSK3*β*), affected mitochondrial transport (anterograde decrease and retrograde increase) rates [85].

The effects of pathological forms of tau on mitochondrial axonal transport were evaluated [86]. Studies in mouse cortical neurons expressing unphosphorylated (Ala mutant, 3A) and a constitutive phosphorylated construct (Asp mutant, 3D) showed mitochondrial movement impairment in 3D positive neurons compared to 3A expressing neurons [86]. In addition, complementary studies of Quintanilla et al. [49] examined the effect of pseudophosphorylated tau at Ser396 and Ser404 on mitochondrial transport in cortical neurons [49]. PHF-1 represents phosphorylated tau at S396/S404 that forms the NFTs in AD neurons [1]. Expression of pseudophosphorylated tau did not affect mitochondrial velocity and movement compared to full-length tau or GFP-expressing neurons [49].

Studies in transgenic* Drosophila* flies expressing human tau demonstrated that the loss of axonal mitochondria produced by genetic ablation of Milton increases tau phosphorylation at an AD-relevant site [78]. In addition, LaPointe et al. [87] using isolated squid axoplasm and monomeric or filamentous forms of human tau demonstrate that tau filaments selectively inhibited anterograde fast axonal transport, triggering the release of conventional kinesin from axoplasmic vesicles through activation of PP1 and GSK3*β* [87]. Interestingly, studies in rTg4510 mice showed altered mitochondrial distribution in presence of tau aggregates and doxycycline treatment (that reverse tauopathy) restored mitochondrial distribution in cultured neurons from rTg4510 mouse [88].

Finally, new studies link the possible role of SNPH in mitochondrial trafficking and neurodegeneration [89]. Cultured hippocampal neurons of SNPH knock-out mice showed increase in mitochondrial motility, which increased the synaptic function [89]. Moreover, SNPH plays an essential role in increasing mitochondrial stationary size in demyelinated axons, and in SNPH deficient axons increased axonal degeneration and neuronal loss [90].

## 5. Improving Mitochondrial Health in AD

In this review, we discussed the importance of mitochondrial dysfunction in the pathogenesis of AD. Mitochondrial injury could affect neuronal function at different levels, synaptic dysfunction being one of the main reasons for memory loss and cognitive impairment in AD [4, 5]. Several groups have suggested improving mitochondrial function, as a valid target to prevent neurodegeneration in AD [91]. These strategies include prevention of mitochondrial fragmentation, reduce ROS levels, increase ATP production, and increase mitochondrial transport [91]. For instance, a study that explored the contribution of mitochondrial dynamics in PD showed that the microinjection with the Drp1-dominant negative K38A restored dopamine release from nigral dopaminergic neurons [91]. This was also observed in C57Bl/6 mice, in which impairment of fission by K38A reduced cell death after treatment with the neurotoxin mPTP [91]. In addition, the treatment of Pink1(−/−) mice with mitochondrial division inhibitor-1 (Mdivi-1), an inhibitor of Drp-1, prevented mitochondrial fragmentation and restored dopamine release in dopaminergic neurons [91]. In addition, pretreatment of hippocampal neurons with Mdivi-1 prevented neuronal death and reduced brain damage after ischemia in a mouse model of epilepsy [92]. Complementary studies with P110, another Drp1 inhibitor, showed inhibition of mitochondrial fission and reduced ROS levels, in cultured neurons that presented mitochondrial fragmentation and oxidative stress [92].

Experiments in neuronal cells lines exposed to oxidative stress showed that the treatment with piracetam, a metabolic enhancer drug, prevented mitochondrial depolarization and increased ATP production [93]. Interestingly, in mouse models that overexpress APP, piracetam reduced A*β* levels and the area of A*β* plaque [93]. Also, piracetam prevented mitochondrial fragmentation in HEK cells treated with the mitochondrial complex I inhibitor, rotenone [93]. Finally, piracetam partially prevented changes in mitochondrial morphology induced by A*β* in SHY5Y cells by switching the mitochondrial fission/fusion cycle from fission to fusion [93]. In addition, studies in neuronal cell lines showed that inhibition of extracellular receptor kinase (ERK1/2) prevented mitochondrial dysfunction induced by high glucose treatment [94, 95]. This is interesting because ERK1/2 controls the activation of Drp1 by its phosphorylation at Ser616 [94, 95]. Further studies explored the role of mitochondrial permeability transition pore (mPTP) on mitochondrial injury in AD [96]. Interestingly, genetic ablation of voltage-dependent anion channel (VDAC1), a component of mPTP, resulted in a decrease in the expression of APP, tau, and PS1 [96]. At the same time, reduction of VDAC1 decreased the activity of Drp1 in neuronal cells [96]. These modifications reduced mitochondrial fission and prevented neuronal death, suggesting that this protein could be a future target for drug development against AD [96].

Several groups have explored different mechanisms to prevent mitochondrial transport defects in AD [97]. For instance, studies in hippocampal neurons treated with tubacin, an inhibitor of HDAC6, showed an increment in mitochondrial traffic [97]. In addition, hippocampal neurons from Hdac6/APPPS1-21 mice (Hdac6 knock-out mice crossed with the double transgenic APPPS1-21) showed that the reduction of HDAC6 restored memory impairment and *α*-tubulin acetylation [97]. This evidence suggests that loss of Hdac6 is protective against A*β*-induced mitochondrial transport defects [98]. Furthermore, treatment with SS31, a mitochondrial-targeted antioxidant, prevented mitochondrial transport decrease in primary neurons from Tg2576 mice [99]. Treatment with SS31 reversed both the trafficking deficit and the occurrence of excess in mitochondrial fission, being a promising molecule to test in AD patients [99]. In the same context, Mao et al. [100] examined the effects of mitochondria-targeted antioxidant catalase (MCAT) against A*β* toxicity in a generated mouse that express MCAT/A*β*PP [100]. Overexpression of MCAT significantly reduced the levels of full-length APP, A*β* levels (40 and 42), A*β* deposits, and oxidative DNA damage compared to A*β*PP mice [100]. Furthermore treatment of cortical neurons with antioxidants such as N-acetylcysteine, vitamin E, or decreasing tau levels improved axonal mitochondrial transport in neurons with a reduced AFG3L2 (a mitochondrial protease related with neurodegenerative disease) expression [101]. On the other hand,* in vivo* deletion of Afg3l2 leads to tau hyperphosphorylation and activation of ERK kinases in cultured neurons [101].

Evidence discussed above indicates that mitochondrial injury participates in the pathogenesis of AD. At the same time, boosting mitochondrial health using fission/fusion inhibitors, as well as mitochondrial-targeted antioxidants, prevented neurodegeneration in AD [91, 92, 102]. However, recent studies have investigated whether defects in mitochondrial biogenesis contribute to mitochondrial abnormalities in AD [102]. Peroxisome proliferator-activated receptor gamma-coactivator 1*α* (PGC-1*α*) [103, 104] and the nuclear factor erythroid 2-related factor (Nrf2) [105] are considered the master pathways that control mitochondrial biogenesis in the brain [102–104]. These pathways control the expression of mitochondrial proteins involved in ETC and antioxidant enzymes that prevent excessive ROS production [103–105]. In this context, Sheng et al. [102] showed that expression of PGC1-*α* and Nrf2 decreased in AD patients and APPswe M17 cells [102]. More importantly is that overexpression of PGC1-*α* prevented mitochondrial biogenesis detriment and mitochondrial dysfunction in APPswe M17 cells [102]. At the same time, several groups have studied the actions of Nfr2 against mitochondrial dysfunction and neurodegeneration [105–110]. Oxidative stress and mitochondrial injury can activate the Nrf2-ARE pathway inducing the expression of several protective enzymes and antioxidants compounds [105]. In addition, the Nrf2-ARE pathway may play a role in the pathogenesis of AD [107]. In this context, recent studies showed reduced levels of Nrf2 in AD brain [107]. Moreover, Nrf2-ARE pathway is inhibited in the APP/PS1 mice, a transgenic AD mice that express APP and presenilin 1 [106]. Further studies showed that Nrf-2 overexpression protected cultured neurons from A*β*-induced neurotoxicity through the increase of mitochondrial biogenesis [106, 107]. Furthermore, neuroprotective effects of Nrf2 have been studied using different natural compounds that activate this pathway [108]. For instance, the use of curcumin and pyrrolidine dithiocarbamate, two electrophilic compounds with the capability to activate Nrf2, ameliorated cognitive defects in transgenic animals that model AD [108]. More importantly, the contribution of Nrf2 to tau pathology in AD was studied [110]. These studies showed that hyperphosphorylated tau accumulated in the brain of Nrf2 knockout mice [110]. In addition, activation of the Nrf2 pathway with sulforaphane, a compound that is present in green broccoli, reduced the levels of phosphorylated tau by induction of autophagy in cortical neurons [110].

**Figure 2:**
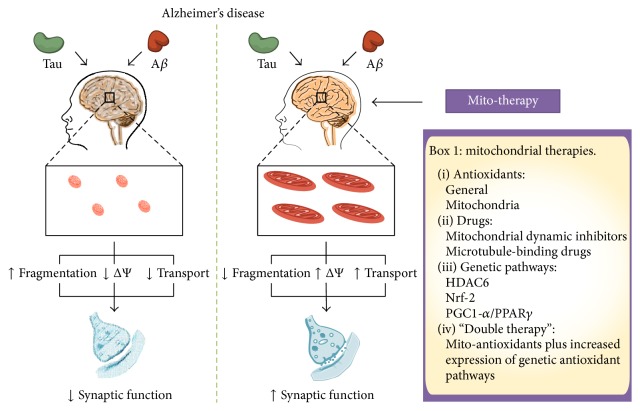
Improving mitochondrial health in AD. In AD, the action of tau and A*β* generates impairment of the mitochondrial function causing fragmentation, depolarization, oxidative stress, and defects in axonal transport. Several strategies have been used to reduce mitochondrial failure in AD. These elements include antioxidants (systemic and mitochondria-targeted), inhibitors of mitochondrial dynamics, microtubules stabilizing drugs, and increase of mitochondrial biogenesis. Also, in this review, we propose the use of a “double mitochondrial therapy,” which means the combinatory use of mitotargeted antioxidants and activators of mitochondrial biogenesis. The use of these therapies can potentially reduce the mitochondrial fragmentation improving the mitochondrial network, restore the membrane potential (increasing ATP production and reducing ROS levels), and increase axonal transport. ΔΨ: mitochondrial membrane potential; VDAC1: voltage-dependent anion channel; HDAC6: histone deacetylase 6; Nrf2: nuclear factor erythroid 2-related factor 2; PGC1-*α*: peroxisome proliferator-activated receptor gamma-coactivator 1 alpha.

Despite the potential benefits of choosing one pathway or another to improving mitochondrial injury in AD, the use of a different strategy that simultaneously activates mitochondrial function by two pathways could have more positive effects. For instance, the use of scavenger's compounds or specific antioxidants for mitochondria showed an important reduction in ROS levels and partially recovered mitochondrial function in neurons [99]. However, if we consider that some elements that control mitochondrial biogenesis contributed to mitochondrial impairment in AD, the use of antioxidants will not correct this deficiency. At the same time, preventing mitochondrial dysfunction through activation of PGC1-*α* and/or Nrf2 pathways requires a complex regulation with no immediate results [102, 105]. In this scenario, the use of mitochondrial antioxidants in combination with activators of mitochondrial biogenesis could have more promising results (Figure 2) [110, 111]. These observations rest on studies discussed in this review, which indicates that mitochondrial dysfunction could be responsible for the neurodegeneration in AD by affecting energy supply and reducing antioxidant defenses. Moreover, mitochondrial injury could participate in the establishment of A*β* and tau pathology, two hallmarks in the pathogenesis of AD [93] (Figure 2). However, further studies are needed to explore this strategy as a valid target to prevent mitochondrial injury in AD.

## 6. Conclusions

In this work, we discussed evidence that indicates the importance of mitochondrial impairment in the pathogenesis of AD. In AD, mitochondrial function could be affected at three distinct levels: (1) structure or morphology, (2) bioenergetics (ATP and oxidative stress), and (3) transport (Figure 1). Changes in morphology could lead to shortened and fragmented mitochondria with energy and antioxidant deficits. Alterations in ATP production and antioxidant defenses will produce inefficient mitochondria, which will be unable to deliver energy supply for neuronal function. Mitochondrial transport is vital for the establishment of neuronal polarity and defects in this process could affect neuronal communication through synaptic process. In general, the impairment of each of these levels could contribute to neuronal injury reported in AD. A*β* aggregates and tau pathology could specifically damage mitochondrial function compromising neuronal viability and communication. Interestingly, complementary studies in neuronal cells and AD mice models suggest that mitochondrial injury is present at early times of the disease. Therefore, rescuing mitochondria through the improvement of mitochondrial dynamics, bioenergetics, and transport should be seriously considered as a valid target for an early therapeutic intervention against neurodegeneration observed in AD (Figure 2).
